# Rapid evolution of promoters from germline-specifically expressed genes including transposon silencing factors

**DOI:** 10.1186/s12864-024-10584-9

**Published:** 2024-07-08

**Authors:** David W. J. McQuarrie, Azad Alizada, Benjamin Czech Nicholson, Matthias Soller

**Affiliations:** 1 School of Biosciences, College of Life and Environmental Sciences, University of Birmingham, Edgbaston, Birmingham, B15 2TT UK; 2 Birmingham Centre for Genome Biology, University of Birmingham, Edgbaston, Birmingham, B15 2TT UK; 3 Cancer Research UK Cambridge Institute, University of Cambridge, Li Ka Shing Centre, Cambridge, CB2 0RE UK

**Keywords:** Piwi-interacting RNA (piRNA), Germline transposon silencing, RNA transgenerational inheritance, Rapid evolution, Promoter evolution, Speciation, Nuclear pore complex, Neuronal wiring, Transposon silencing

## Abstract

**Background:**

The piRNA pathway in animal gonads functions as an ‘RNA-based immune system’, serving to silence transposable elements and prevent inheritance of novel invaders. In *Drosophila*, this pathway relies on three gonad-specific Argonaute proteins (Argonaute-3, Aubergine and Piwi) that associate with 23–28 nucleotide piRNAs, directing the silencing of transposon-derived transcripts. Transposons constitute a primary driver of genome evolution, yet the evolution of piRNA pathway factors has not received in-depth exploration. Specifically, channel nuclear pore proteins, which impact piRNA processing, exhibit regions of rapid evolution in their promoters. Consequently, the question arises whether such a mode of evolution is a general feature of transposon silencing pathways.

**Results:**

By employing genomic analysis of coding and promoter regions within genes that function in transposon silencing in *Drosophila*, we demonstrate that the promoters of germ cell-specific piRNA factors are undergoing rapid evolution. Our findings indicate that rapid promoter evolution is a common trait among piRNA factors engaged in germline silencing across insect species, potentially contributing to gene expression divergence in closely related taxa. Furthermore, we observe that the promoters of genes exclusively expressed in germ cells generally exhibit rapid evolution, with some divergence in gene expression.

**Conclusion:**

Our results suggest that increased germline promoter evolution, in partnership with other factors, could contribute to transposon silencing and evolution of species through differential expression of genes driven by invading transposons.

**Supplementary Information:**

The online version contains supplementary material available at 10.1186/s12864-024-10584-9.

## Background

Eukaryotic organisms are continuously challenged from genomic parasites called transposable elements (TEs) [1–4]. Unchecked transposon activity often results in reduced reproductive fitness [5–8]. To negate the detrimental effects posed by TE mobilisation, vital regulatory pathways evolved that efficiently suppress transposon activity. At the centre of these are 23–28 nucleotide small RNAs, called PIWI-interacting RNAs (piRNAs). piRNAs are generated from transposons and from genomic clusters and bind to Argonaute proteins of the PIWI-clade, including P-element induced wimpy testis (Piwi). Aubergine (Aub) and Argonaute-3 (Ago3). The piRNA pathway is often referred to as an ‘RNA-based immune system’ thanks to its ability to adopt to new TE insertions and the memory of past transposon activity through piRNA clusters through RNA transgenerational inheritance [1, 2, 9, 10].

**Fig. 1:**
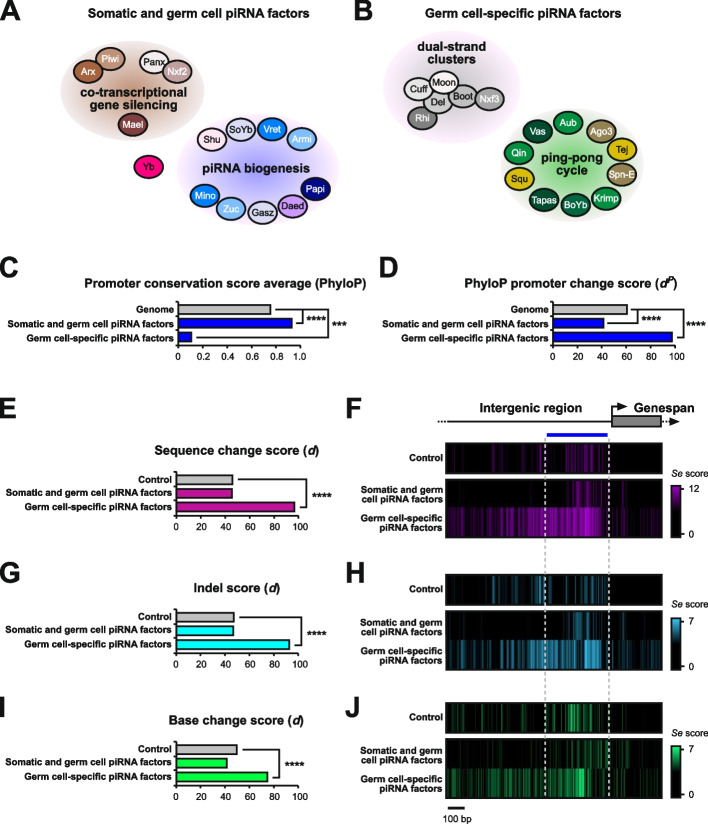
Promoter regions of germ cell-expressed piRNA processing factors are hot spots for rapid evolution. **A** and **B** Schematic depiction of somatic and germ cell piRNA factors involved in co-transcriptional gene silencing and piRNA biogenesis (**A**) and germline-specific piRNA pathway genes involved in dual-strand cluster regulation and the ping-pong cycle (**B**) in the *Drosophila* ovary. **C** and **D** PhyloP27way conservation score averages (**C**) and PhyloP27way promoter change *d*^*P*^scores (**D**) for the 350-nucleotide promoter regions of the somatic and germ cell piRNA factors and the germ cell-specific piRNA factors compared to all genes in the *Drosophila* genome. Statistically significant differences from unpaired student t-tests (**C**) and non-parametric chi-squared tests (**D**) are indicated by asterisks (*** *p*≤0.001, **** *p*≤0.0001 following Bonferroni correction). **E**-**J** Heatmaps indicating sequence (F, purple), indel (H, blue) or base change (J, green) accumulation, and their quantification (E-I) depicted as *d* scores for each of the analysed gene groups compared to the control among closely related *D. melanogaster*, *D. simulans*, *D. sechellia*, *D. yakuba* and *D. erecta*. Regions of 1000 nucleotides upstream and 300 nucleotides downstream of the TSS were analysed. Analysis was performed for the control group (m^6^A writer complex and readers), the somatic and germ cell piRNA factors, and the germ cell-specific piRNA factors. The blue line indicates the promoter region used for quantification of the substitution rate based on the gene and intergenic region schematic. Statistically significant differences from non-parametric chi-squared tests are indicated by asterisks (**** *p*≤0.0001 following Bonferroni correction)

The piRNA pathway is arguably best understood in the ovary of *Drosophila melanogaster*. It was shown that ovaries feature two branches of the silencing machinery [5], one that is active in germ cells and a condensed version that is specific to somatic follicle cells that surround the germline [2, 11]. Somatic follicle cells only feature unistrand piRNA clusters, such as *flamenco* (*flam*), and exclusively produce piRNAs via a Zucchini (Zuc)-dependent biogenesis mechanism that takes place on the mitochondria surface involving Armitage (Armi), Papi, Vreteno (Vret), Gasz, Daedalus (Daed), Minotaur (Mino), Shutdown (Shu), and Sister of Yb (SoYb) (Fig. 1A) [3, 12, 13]. Licensing of *flam* for piRNA production takes place upstream, at so-called Yb-bodies [1, 12]. Somatic cells only express Piwi, which following the association with a mature piRNA shuttles to the nucleus. There, Piwi-piRNA complexes scan for nascent TE transcripts and instruct co-transcriptional gene silencing (cTGS) that requires components of the general chromatin silencing machinery and the piRNA pathway-specific factors Asterix (Arx), Piwi, Panoramix (Panx), Nuclear export factor 2 (Nxf2) and Maelstrom (Mael) (Fig. 1A) [1, 2, 12, 14].

In germ cells, piRNAs predominantly originate from dual-strand clusters and are produced by a different biogenesis mechanism called the ping-pong amplification cycle (Fig. 1B) [1–3, 5, 11, 12, 15]. Dual-strand clusters harbour remnants of transposons and are expressed via a non-canonical transcription process that is centred on a complex containing the HP1a homolog Rhino (Rhi), Deadlock (Del) and Cutoff (Cuff), and further requires Moonshiner (Moon) (Fig. 1B). This Rhi-Del-Cuff complex also recruits a non-canonical export machinery consisting of Bootlegger (Boot) and Nuclear export factor 3 (Nxf3), which transport piRNA precursors for processing to a germ-cell specific perinuclear region termed “nuage” (Fig. 1B). Here, the ping-pong cycle takes place and relies on the two PIWI proteins Aubergine (Aub) and Argonaute 3 (Ago3), as well as several RNA binding proteins and Tudor-domain containing factors including Tejas (Tej), Vasa (Vas), Qin, Squash (Squ), Tapas, Brother of Yb (BoYb), Krimper (Krimp), and Spindle-E (Spn-E) (Fig. 1B) [1–3, 12, 15, 16].

Channel nuclear pore proteins (Nups) have been implicated in piRNA regulated transposon silencing in *C. elegans* [17]. Interestingly, in *Drosophila*, Nups were shown to affect transposon silencing in both the germline [16, 18], and in somatic cells of the ovary, here specifically by contributing to the conversion of precursor RNAs from the *flam* locus into piRNAs [19]. Recently, we found that inner and outer ring Nups in the nuclear pore complex (NPC) evolve rapidly through indel accumulation in their promoters [20, 21]. Such promoter indel variability in *Nup54* has dominant, pleiotropic effects on sexual differentiation including neuronal wiring important for female post-mating behaviours directed by male-derived sex peptide, that as a result of sexual conflict could drive speciation by such mechanism [17, 20, 22]. Intriguingly, nuclear import/export pathways have been linked to speciation through hybrid incompatibility, but are in addition important for many cellular processes beyond piRNA processing [23, 24]. Hence, promoter evolution particularly of piRNA processing genes could have wide impact on transposon silencing and evolution of species through differential expression of genes, but whether regulators of germline transposon silencing generally evolve rapidly through their promoters has not been determined.

Here, we analyse the promoters of genes involved in the ovarian piRNA pathway. We compare piRNA factors required in somatic cells (Yb) or in both the somatic and germline compartments (cTGS and piRNA biogenesis, Fig. 1A) with those that are germ cell-specific (dual-strand clusters and the ping-pong cycle, Fig. 1B). We find that the promoters of germline-specific piRNA factors in insects are hot spots for rapid evolution, while piRNA factors expressed in both somatic and germ cells evolve slower. Analysis of genes with significant accumulation of promoter changes reveals a mix of indel and base change accumulation. Further, our analysis reveals that rapid promoter evolution is a general feature of genes specifically expressed in germ cells, while soma-expressed genes evolve at a similar rate to the average of all *Drosophila* genes. Through cross-species differential ovary RNA-seq analysis we reveal that germ cell-specific genes minimally diverge in gene expression levels compared to genes expressed in somatic cells. Our findings highlight that promoters of germline genes involved in transposon silencing evolve rapidly and are accompanied by diverging gene expression, suggesting a possible mode of rapid speciation through accumulation of changes in promoters in correlation with additional regulatory factor divergence.

## Results

### Promoter regions of germline-specific piRNA factors are hot spots for rapid evolution

Since Nups have been shown to function in transposon silencing in the germline (12 from 14 tested are above the control) and display fast evolving promoters [16, 20, 21], we analysed the rate of evolution in promoters and coding regions of piRNA pathway genes. We separated all piRNA pathway genes based on their genetic requirement into two categories: piRNA factors essential in somatic cells, including the soma-specific piRNA factor Yb and piRNA factors expressed in both somatic and germline cells (cTGS and piRNA biogenesis, Fig. 1A), and germ cell-specific piRNA proteins (dual-strand clusters and ping-pong cycle, Fig. 1B). The piRNA factors essential in somatic cells include *armi*, *papi*, *vret*, *Gasz*, *daed*, *mino*, *zuc*, *shu*, *SoYb*, *Yb*, *arx*, *piwi*, *panx*, *nxf2*, and *mael* (Fig. 1A). The group of piRNA factors that are exclusively required in the germline are *aub*, *tej*, *Ago3*, *vas*, *qin*, *squ*, *tapas*, *BoYb*, *krimp*, *spn-E, Boot*, *moon*, *nxf3*, *cuff*, *rhi*, and *del* (Fig. 1B) [3, 14, 25].

We defined the promoter containing region as a 350-nucleotide (-30 to -380) window upstream of the predicted TATA box region 30 nucleotides upstream of the transcription start site (TSS) [26]. In a first approach, we performed a genome-wide promoter evolution analysis of germline-specific as well as somatic and germ cell piRNA factors and compared it to all genes in the *Drosophila* genome (Fig. 1C and D). We used publicly available PhyloP27way data as the conservation score. We observed increased conservation in promoter regions of piRNA factors expressed in somatic and germ cells, and lower conservation of germ cell-specific piRNA factors compared to the genome (Fig. 1C). Quantifying accumulation of changes revealed that germ cell-specific piRNA factors had accumulated significantly more sequence changes in promoter regions compared to the genome (Fig. 1D). Of note, the somatic and germ cell piRNA factor group evolved slower compared to the genome (Fig. 1D).

We next compared indel and base changes in the promoter regions between five closely related *Drosophila* species (*D. melanogaster*, *D. simulans*, *D. sechellia*, *D. yakuba* and *D. erecta*). Promoter scores (*d*) were calculated and compared to a control group comprised of the m^6^A methylation machinery genes (*Mettl3*, *Mettl14*, *fl(2)d*, *virilizer*, *flacc*, *nito* and *Hakai*, *Ythdc1* and *Ythdf*), chosen due to their high evolutionary conservation and requirement for strict stoichiometry for functionality, making this an ideal control group to monitor promoter evolution [21, 27, 28]. In this analysis, the group of somatic and germ cell piRNA factors showed similar evolution rates compared to the control (Fig. 1E-J), while germ cell-specific piRNA factors evolved at a higher rate (Fig. 1E and F), accruing a significantly increased number of promoter-located indels (Fig. 1G and H) and base changes (Fig. 1I and J). At the individual gene level, 7 out of 14 germ cell-specific piRNA factors showed significant accumulation of promoter changes (Supplementary Fig. 1A). *Ago3* was omitted due to its low conservation between closely related *Drosophila* species [29–31]. Among the somatic and germ cell piRNA factors, 3 out of 15 genes had significant accumulation of promoter changes (Supplementary Fig. 1A).

To assess general evolution in the coding regions, we used McDonald-Kreitman tests (MKTs) and analysed polymorphisms and divergence within *D. melanogaster* and between *D. melanogaster* and *D. simulans* for two ancestral populations (Congo and Zambia) [32]. This analysis indicates that germ cell-specific piRNA factors are under positive selection (Supplementary Fig. 1B). Conversely, piRNA factors expressed in both somatic and germ cells were not under positive selection (Supplementary Fig. 1B).

### Promoters of genes required for germline transposon silencing are fast evolving

**Fig. 2:**
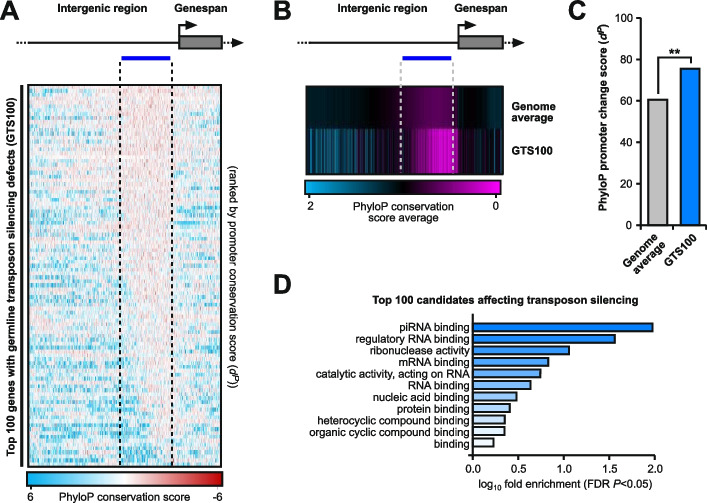
Promoters of genes required for germline transposon silencing evolve fast. **A** PhyloP27way nucleotide scores for the top 100 genes affecting transposon silencing in germ cells (GTS100) based on transposon derepression *z* scores and ordered by promoter conservation *d*^*P*^ scores. A region of 1000 nucleotides upstream and 300 nucleotides downstream are shown. Red represents lower conservation while blue represents higher. **B** Average PhyloP27way nucleotide scores for the GTS100 genes affecting transposon silencing in germ cells compared to the genome average. **C **Comparison of PhyloP27way promoter change scores (*d*^*P*^) for each of the GTS100 genes affecting transposon silencing in germ cells compared to the genome average. Statistically significant differences from non-parametric chi-squared tests are indicated by asterisks (** *p*≤0.01 following Bonferroni correction). **D** Representative GO analysis for the GTS100 gene candidates affecting transposon silencing

Given the rapid evolution of germ cell-specific piRNA factors among closely related *Drosophila* species, we wanted to determine (1) whether this is a general feature of insect evolution, and (2) whether this is a general feature of genes required for transposon silencing with pleiotropic roles that have been identified in a genetic screen (GTS100 genes), since this provides a larger gene group [16]. To get a scale of the persistence of evolutionary changes in promoters of GTS100 genes we used publicly available PhyloP27way data from UCSC genome browser [33, 34]. Genes were ordered based on transposon de-repression scores [16] and *d*^*P*^ was calculated for individual genes, the genome average as a control group, and for the average of GTS100 gene promoter regions (Fig. 2A-C, 3A). Genes without full PhyloP coverage were removed from the analysis (where ≥ 1 nucleotide of the analysed genomic region contained the *D. melanogaster* sequence only).

**Fig. 3:**
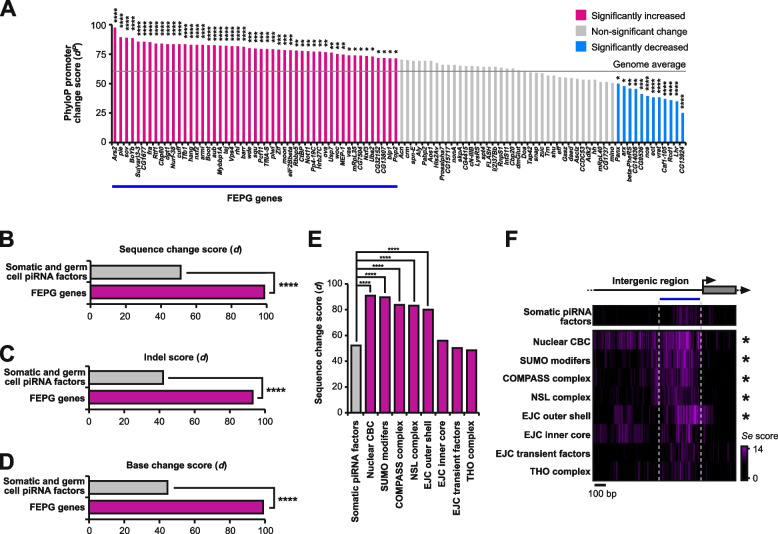
Promoters of FEPG genes and their associated complexes are hotspots for rapid evolution. **A** Individual gene comparison of PhyloP27way promoter change scores (*d*^*P*^) for GTS100 genes affecting transposon silencing in germ cells compared to the genome average. Statistically significant differences from non-parametric chi-squared tests are indicated by asterisks (* *p*≤0.05, ** *p*≤0.01, *** *p*≤0.001, **** *p*≤0.0001 following FDR correction). **B**-**D** Sequence analysis for change accumulation among closely related *D. melanogaster*, *D. simulans*, *D. sechellia*, *D. yakuba* and *D. erecta*. Comparisons were performed for sequence change (**B**), indel (**C**), and base change (**D**) *d* scores for the significantly increased GTS100 genes in the FEPG gene group compared to the somatic and germ cell piRNA factors. Statistically significant differences from non-parametric chi-squared tests are indicated by asterisks (**** *p*≤0.0001 following Bonferroni correction). **E** Comparison of *d* scores for protein complex genes involved in germ cell transposon silencing compared to the somatic and germ cell piRNA factors. Statistically significant differences from non-parametric chi-squared tests are indicated by asterisks (**** *p*≤0.0001 following Bonferroni correction). **F** Heatmaps indicating sequence change accumulation for protein complex genes involved in germ cell transposon silencing compared to the piRNA factors shown in purple among closely related *D. melanogaster*, *D. simulans*, *D. sechellia*, *D. yakuba* and *D. erecta*. Regions of 1000 nucleotides upstream and 300 downstream of the TSS were analysed. The blue line indicates the promoter region used for quantification of the substitution rate

The promoter change score average of GTS100 genes showed a significant increase in promoter nucleotide changes compared to the control (Fig. 2C). Although many of these genes have additional roles, gene ontology (GO) analysis confirmed a primary role in piRNA binding (Fig. 2D) [35–37].

To understand whether GTS100 genes were individually fast evolving, individual *d*^*P*^ scores were calculated (Fig. 3A). Compared to the genome average, 49 genes (FEPG, Fast Evolving Promoter Germline genes) were significantly increased in promoter change events, while 12 were significantly decreased (Fig. 3A). Of note *Brother of Yb* (*BoYb*) which is considered the germline replacement of *Yb* [3], also displayed a fast evolving promoter in contrast to its somatic counterpart *Yb* (Fig. 3A). To specifically look at indels and base changes at a high confidence level, we analysed FEPG genes for rapid promoter evolution between the previously used five *Drosophila* species and compared them to the somatic and germ cell piRNA factor control group (Fig. 3B-D). Here, indels and base changes were measured, showing a significant increase in both indels and base changes compared to the control (Fig. 3C and D).

### A subset of piRNA factors that are part of distinct protein complexes display hot spots for rapid evolution in promoters

In a saturating genetic screen for regulators of transposon silencing in germ cells members of the Nup complex were identified as regulators of germline piRNA silencing, and later shown to rapidly evolve through their promoters [16, 20, 21]. In the same screen, numerous genes whose products are part of distinct protein complexes were identified, including the nuclear cap-binding complex (CBC), THO complex, the exon junction complex (EJC) inner core, outer shell and transient factors, the non-specific lethal (NSL) complex, SUMOylation modifiers and the complex of proteins associated with Set1 (COMPASS) (Supplementary Fig. 2). Given the fast evolution of promoters in germline-specific piRNA pathway genes and the Nup complex, we analysed promoters of the additional protein complexes affecting germline piRNA silencing to see whether their rate of evolution matched that of other piRNA silencing factors. Compared to somatic and germ cell piRNA factors, promoter regions for the nuclear CBC, SUMO modifiers, COMPASS complex, NSL complex and EJC outer shell showed a significant accumulation of sequence changes (Fig. 3E and F). Analysis of individual genes from both significant and non-significant gene groups revealed that 43% of analysed genes have rapidly accumulated sequence changes in their promoters (Supplementary Fig. 2). Specifically looking at genes in fast evolving complexes (Fig. 3E and F) revealed that 56% of genes in these groups rapidly evolve through accumulation of mutations in their promoters (Supplementary Fig. 2).

When analysing the base changes and indels of rapidly evolving promoters (Supplementary Fig. 3A-D), indel accumulation was observed for the SUMO modifiers, COMPASS complex, NSL complex and EJC outer shell (4 out of 5), while the nuclear CBC showed a significant decrease (Supplementary Fig. 3B). The nuclear CBC, SUMO modifiers and COMPASS complex also showed a significant accumulation of base changes in their promoter regions (3 out of 5) (Supplementary Fig. 3D). Individually, 4 out of 27 genes were significantly enriched in indel events (*Hcf*, *dgt1*, *nsl1*, and *Bin1*), while base change accumulation events indicated 8 out of 27 genes (*Ars2*, *cbp80*, *Ulp1*, *Set1*, *Hcf*, *Cfp1*, *MBD-R2*) being significantly increased (Supplementary Fig. 3E).

Next, we analysed the rate of evolution in the coding regions of these complexes using MKT tests [32]. This analysis flagged the EJC outer shell, EJC transient factors, NSL complex, and SUMO modifiers as under positive selection in both populations, while the Nuclear CBC was under positive selection in the Zambia population only (Supplementary Fig. 4).

### Analysis of piRNA factor differential gene expression in ovaries of *D. melanogaster* and *D. yakuba*

**Fig. 4:**
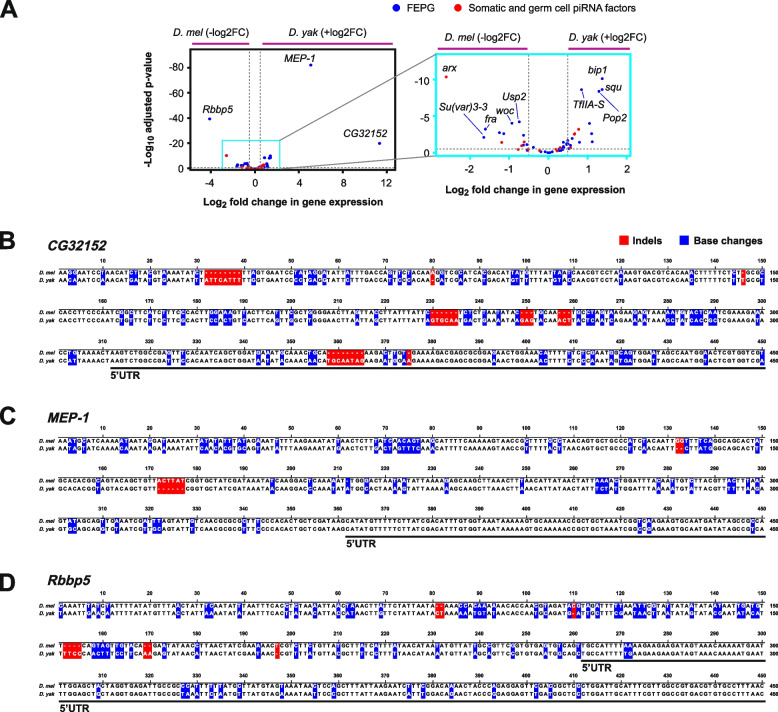
Analysis of differential gene expression of FEPG genes in *D. melanogaster* and *D. yakuba* ovaries. **A** Volcano plot showing differential gene expression (DESeq2) between *D. melanogaster *and *D. yakuba *ovaries (RNA-seq data; Supplementary Dataset 1) for FEPG (blue) and the somatic and germ cell piRNA factors (red). Dashed lines indicate significance thresholds (log_10_ adjusted *p* value <-0.5, log_2_ fold change >0.5/<-0.5). **B**-**D** Alignment of the promoter regions of *CG32152, MEP-1* and *Rbbp5* for *D. melanogaster* and *D. yakuba* with indels (red) and base changes (blue) indicated as boxes. The black lines indicate gene 5’ UTRs

Since promoters of FEPG genes are fast evolving, we analysed publicly available RNA-seq data from ovaries between *D. melanogaster* and *D. yakuba* to assess gene expression divergence in the FEPG gene group compared to the somatic and germ cell piRNA factors (Fig. 4A) [38–40]. Here, many FEPG genes had a tendency for slight significant divergence in gene expression in both directions (*D. melanogaster* or *D. yakuba*), but this was true for only few somatic and germ cell piRNA factors genes including *arx* (Fig. 4A). Notably, we observed large expression divergence for *CG32152, MEP-1* and *Rbbp5* (Fig. 4A). Aligning the promoters of these three genes revealed an increased number of indels and base changes compared to the 5’ UTR or the coding region (Fig. 4B-D), but the cohort was unfortunately too small to detect whether increased changes in promoters correlate with altered expression between the two species. Further limitations stem from the pleiotropic roles of the analysed genes, and therefore expression profiles likely differ across cell types in the analysed tissue.

To further analyse the promoters of these genes we performed motif enrichment analysis indicative of transcription factor binding sites in promoters of the somatic and germ cell piRNA factors (Fig. 1A), germ cell-specific piRNA factors (Fig. 1B), and the FEPG piRNA factor gene groups (Supplementary Fig. 5). To contextualise functionality of the enriched motifs we analysed publicly available female adult *D. melanogaster* ovary expression data. Of these, transcription/DNA binding factors Mothers against dpp (Mad), Boundary Element-Associated Factor of 32kD (BEAF-32) and DNA replication-related element factor (Dref) were expressed in ovaries and their binding motifs were enriched in the FEPG piRNA factor gene group (Supplementary Fig. 5) [41–44].

### Germ cell-specific genes show rapid evolution of promoters associated with divergent gene expression between species

**Fig. 5:**
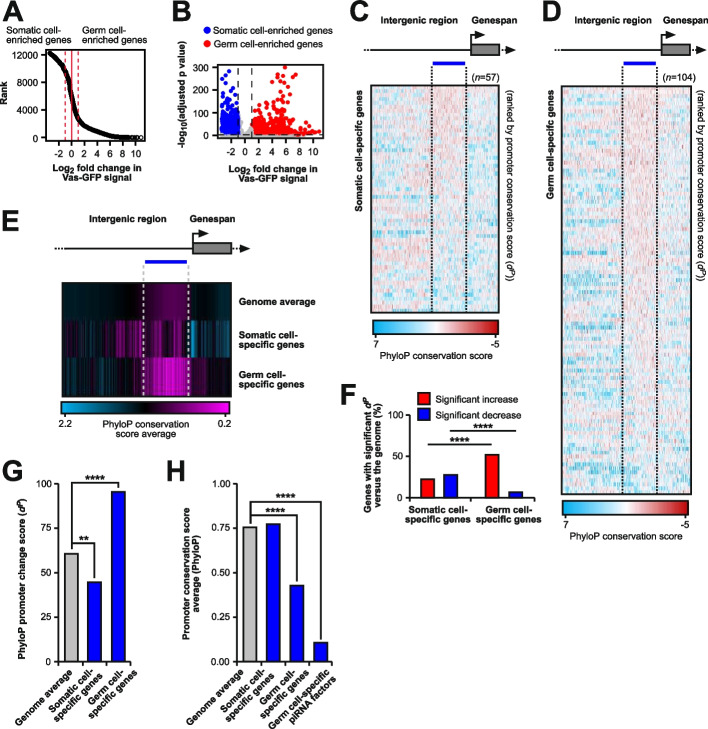
Germ cell-specifically expressed, but not somatic cell-specifically expressed gene promoters evolve rapidly and are accompanied by divergence in gene expression. **A** and **B** Ranking of genes (**A**) and volcano plot showing differential gene expression (**B**) using vasa-GFP signal from FACS-sorted vasa-GFP+/- cells from ovaries of transgenic vas-GFP *D. melanogaster* [45]*.* Groups are defined as germline-enriched (vasa-GFP+) and soma-enriched (vasa-GFP-) genes. **C** and **D** PhyloP27way nucleotide scores for somatic cell-specific (**C**) and germ cell-specific (**D**) expressed genes ordered by promoter change *d*^*P*^ scores. A region of 1000 nucleotides upstream and 300 nucleotides downstream are shown. Red represents lower conservation while blue represents higher. **E** Average PhyloP27way nucleotide scores for all genes in the *Drosophila* genome, somatic cell and germ cell-specific genes. Purple represents lower conservation while blue represents higher. **F** The percentage of somatic cell and germ cell-specific genes with significant promoter nucleotide changes (*d*^*P*^ ) versus all genes in the *Drosophila* genome average, divided by significantly increased (low conservation) or decreased (high conservation). Statistically significant differences from non-parametric chi-squared tests are indicated by asterisks (**** *p*≤0.0001 following Bonferroni correction). **G** and **H** PhyloP27way conservation score averages (**G**) and PhyloP27way promoter change scores (*d*^*P*^) (**H**) for the 350-nucleotide promoter regions compared for all genes in the *Drosophila* genome, somatic cell-specifically expressed and germ cell-specifically expressed genes. Statistically significant differences from unpaired student t-tests (**G**) and from non-parametric chi-squared tests (**H**) are indicated by asterisks (** *p*≤0.01,**** *p*≤0.0001 following Bonferroni correction)

To investigate whether the rapid evolution of promoters in piRNA factors expressed in germ cells is a common feature of germline-expressed genes, we used RNA-seq data from FACS-sorted vasa-GFP ovaries [45] to identify genes specifically expressed in either germ cells or somatic cells of the ovary. First, using differential RNA-seq analysis of FACS-sorted vasa-GFP-positive (germline) and vasa-GFP-negative (somatic) cells from ovaries of a transgenic vas-GFP *D. melanogaster* strain, we defined germline-enriched (vasa-GFP + ; log_2_ fold change > 2, adjusted *p* value < 0.01) and soma-enriched (vasa-GFP-; log_2_ fold change < -1, adjusted *p* value < 0.01) genes (Fig. 5A and B).

Next, using RNA-seq data from ovarian somatic cells (OSCs) (GSE160860) [46], we further filtered the soma-enriched genes that are highly expressed in OSCs. Additionally, we further filtered the germline-enriched genes that show expression in early fly embryos (0-2h, modENCODE). In this way, we generated a stringent list of 107 genes specifically expressed in germ cells and of 59 soma-expressed genes (Supplementary Dataset 1). Of note, the soma-enriched gene group contained no piRNA factors expressed in both somatic and germ cells, while the germline-enriched gene group contained 7 from 107 genes identified as germ cell-specific piRNA factors (Supplementary Dataset 1).

We then analysed sequence change accumulation in promoters using PhyloP data and calculated *d*^*P*^ for all *D. melanogaster* genes and genes expressed specifically in somatic cells or the germline (Fig. 5C and D) or the average (Fig. 5E). Germ cell-specifically expressed genes significantly accumulated changes (*d*^*P*^) in their promoters compared to soma-expressed genes (Fig. 5F). Comparison of the average promoter PhyloP scores and *d*^*P*^ conservation scores between the genome average and somatic or germ cell-specifically expressed genes equally revealed a significant increase in germ cell gene promoter nucleotide changes compared to the genome average (Fig. 5G and H).

**Fig. 6:**
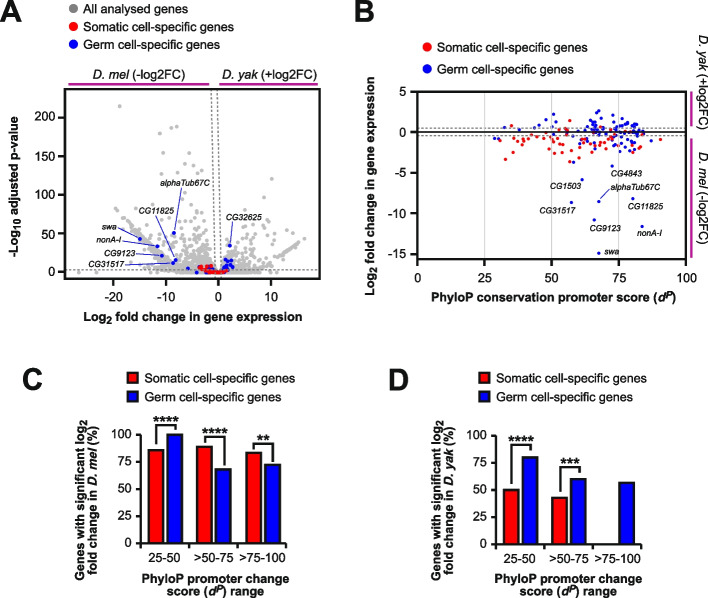
Analysis of gene expression divergence between germ cell and somatic cell-specifically expressed genes. **A** Volcano plot of differential gene expression between *D. melanogaster *and *D. yakuba *ovaries (RNA-seq data; Supplementary Dataset 1) for all (grey), germ cell-specific (blue) and somatic cell-specific (red) gene groups. Dashed lines indicate significance (log_10_ adjusted *p* value <-0.5, log_2_ fold change >0.5/<-0.5). **B** Scatterplot of individual gene *d*^*P*^ scores plotted against fold-change expression differences between *D. melanogaster* and *D. yakuba* ovaries (RNA-seq data; Supplementary Dataset 1) for germ cell-specific (blue) and somatic cell-specific (red) gene groups. The grey dotted lines indicate log_2_ fold change threshold values of >0.5 and <-0.5. **C** and **D** Comparison of the expression distribution and percentage of genes with significant changes in *D. melanogaster* log_2_ fold change (<-0.5, C) or significant changes in *D. yakuba* log_2_ fold change (<0.5, D) expression changes in PhyloP conservation promoter score (*d*^*P*^) ranges >25-50, >50-75, and >75-100. Expression changes for germ cell-specific and somatic cell-specific gene groups were analysed between *D. melanogaster* and *D. yakuba*. Statistically significant differences from non-parametric chi-squared tests are indicated by asterisks (** *p*≤0.01,*** *p*≤0.001, **** *p*≤0.0001 following FDR correction)

Since rapid promoter evolution of germline piRNA silencing factors correlated with gene expression divergence in our previous analysis, we hypothesised that this was a general feature of germ cell genes. To test this, we compared gene expression divergence between somatic cell-specific and germ cell-specific genes using publicly available RNA-seq data of *D. melanogaster* and *D. yakuba* ovaries (Fig. 6A) [38–40]. Notably, genes specifically expressed in germ cells showed slightly higher divergence in expression compared to soma-expressed genes (Fig. 6A). When *d*^*P*^ conservation scores were plotted against the gene expression change between *D. melanogaster* and *D. yakuba*, the germ cell-specifically expressed genes showed significant divergence in expression for all *d*^*P*^ score groups compared to soma-expressed genes, though the distribution of log_2_ fold changes in expression varied significantly for only the > 25–50 and > 50–75 groups (Fig. 6B-D, Supplementary Fig. 6).

## Discussion

Through genomic analysis of germline and somatic transposon silencing genes and their regulators, we identify hot spots for rapid evolution in promoter regions (-30 to -380 nucleotides upstream of the TSS) of germ cell-specific (dual-strand clusters and the ping-pong cycle), but not soma-expressed piRNA pathway genes (cTGS and piRNA biogenesis). Further, we show that rapid promoter evolution is a general feature of germ cell-specific genes compared to those expressed only in the soma. Our analysis suggests that this mode of evolution in the germline pathway could be a general feature of at least insect evolution. Overall, our results point towards a key role for rapid evolution of gene promoters in the germ cell-specific piRNA pathway which could, coupled with other drivers of expression divergence, impact the expression of germline regulatory genes.

Transposon mobility has been attributed to the accumulation of sequence changes in promoter regions because of the presence of open chromatin around the TSS of genes [47]. Interestingly, in core NPC genes, rapid evolution mostly led to accumulation of indels [20, 21], while piRNA pathway genes display equal accumulation of indels and base changes. Likely, indels have more profound effects on changes in transcription than single nucleotide alterations. This feature might reflect that compromised transposon silencing causes sterility, hence not allowing for substantial changes in expression in piRNA processing genes. Likewise, stoichiometric changes in protein complexes can drastically alter complex functionality, e.g. the male-specific lethal (MSL) complex binds less to its targets when one component of the complex is missing [48, 49]. Hence, changes in the protein levels of individual piRNA factors likely have dominant effects on their capacity to silence transposon activity.

The occurrence of novel transposons can fundamentally impact species reproductive success through a phenomenon call hybrid dysgenesis [50]. Here, if a novel transposable element is transmitted by the male, female fertility is severely compromised and can result in sterility as a result of missing piRNA silencing of the novel invader. Essential to combat these novel active transposable elements is the ping-pong cycle, primed by transcripts from the novel transposon. Accordingly, females can be become resistant to such novel transposons over time through RNA transgenerational inheritance of piRNAs to build up ping-pong cycle amplification in germ cells [9, 10, 51].

Our examination of evolution in coding regions flagged germ cell-specific piRNA factors, as well as some pleiotropic piRNA processing associated complexes (EJC outer shell, EJC transient factors, NSL complex, SUMO modifiers, and Nuclear CBC) as under positive selection. This is unsurprising in the case of primary piRNA processing factors in the germline, as there are many examples of these factors being under selection [52–60]. For instance, replacement of *D. melanogaster rhino* and *deadlock* genes with *D. simulans* homologues results in non-functionality [53]. Here, *D. simulans* Rhino binding domains no longer bind to *D. melanogaster* Deadlock, resulting in failed localisation to piRNA clusters [53]. Such effects resulting from few amino acid changes act as powerful driving forces in diverging piRNA processing. In this setting, rapid promoter evolution of piRNA silencing genes could act as an additional factor driving changes in expression levels and factor stoichiometry for piRNA processing divergence, which may be difficult to explore when coupled with functional divergence. Intuitively, one would associate rapid promoter evolution with changes in expression, however, we observed minimal effects on expression. Since transposon silencing is essential, changes in *cis* regulatory elements of these regulatory factors are likely constrained by compensatory mechanisms and could be accompanied by changes in enhancer regions. Whether this is the case would require a detailed knowledge of relevant enhancers and transcription factor binding.

The pattern of changes observed in fast evolving promoters like germline specific piRNA factors and Nups resembles the outcome of a *P*-element mutagenesis experiment in the *egghead* (*egh*) locus coding for a glycosphingolipid biosynthesis enzyme [61]. Here, multiple base changes were observed in the region of the first promoter after *P*-element mutagenesis, but not an actual *P*-element insert, resulting in a sex peptide insensitive allele *egh*^*cm*^. Mutations in the *egh* gene result in pleiotropic phenotypes and the *egh*^*cm*^ allele disrupts neuronal wiring required for the female post-mating response and development of the optic lobe [22, 61, 62].

Since establishing transposon resistance involves forced selection, genes whose expression changes result in pleiotropic effects, like channel Nup54, could combine adaptations in piRNA processing with changes in neuronal wiring resulting in altered behavioural preferences [20]. Of note, in *D. simulans*, a close relative of *D. melanogaster*, projections of *fruitless* P1 sensory neurons that control courtship have changed and alter mate preference [63].

The severe impact of hybrid dysgenesis on fertility likely limits fast evolution to species with high numbers of progeny. Perhaps this can explain the differences seen when comparing insect and mammalian promoters that are generally more conserved [26]. However, rapid promoter evolution has also been observed in primate promoters, suggesting a common mode of evolution that has yet to be explored [20, 21, 26, 64].

## Materials and methods

### Sequence/data retrieval and alignment

Gene and promotor sequences were retrieved from UCSC Genome Browser using the UCSC Table Browser sequence retrieval tool [33, 34]. A standardised region of 2000 bases upstream of the annotated gene TSS was exported for each gene to ensure inclusion of promoter regions. Sequences were imported and aligned with clustalW in MEGA11 [65]. PhyloP27way data were sourced from UCSC genome browser (genome.ucsc.edu) through the Table Browser tool [33, 34]. Data points were collected for a region of 1000 nucleotides upstream and 300 nucleotides downstream for each of the analysed genes. Genes without full species coverage when analysing evolution between the 5 *Drosophila* species, where ≥ 1 species had no conserved genomic sequence were removed from the analysis. Genes without full PhyloP coverage were removed from the analysis (where ≥ 1 nucleotide of the analysed genomic region contained the *D. melanogaster* sequence only). Genes present in more than one group in direct comparisons were removed from that comparison.

### Molecular evolution of open reading frames analysis

Analysis of adaptive protein evolution was performed via MKTs [32] using the PopFly online database (imkt.uab.cat) which uses *Drosophila* Genome Nexus project sequence data [66–68]. Polymorphism and divergence data were collected from the ancestral Congo and Zambia populations, chosen due to their high ancestral stability compared to other populations. The ‘Standard MKT’ test was used to calculate results for individual genes. Fisher’s Exact Test with significance defined as FDR corrected *p* ≤ 0.05 was used to calculate significance between nonsynonymous to synonymous polymorphisms within *D. melanogaster* or between *D. melanogaster* and *D. simulans*.

### Identification of promotor hot spots and comparison of substitution rates

All gene models were manually inspected in FlyBase using the JBrowse browser and dominant transcripts were chosen based on comparative modENCODE expression data [69]. Gene and promoter sequence alignments were trimmed to -1000/ + 300 nucleotides from the TSS of each gene. To calculate the frequency of sequence change hotspots in promoter sequences, we calculated the hotspot accumulation score *d* using alignments generated as described, or PhyloP data. Alignments were translated into ‘events’ for each species compared to *D. melanogaster*, where for each nucleotide in the sequence, 0 signified a conserved sequence and 1 signified a sequence change (base change or indel event) [70]. Events were calculated for all changes, as well as base changes and indels individually. The sum of events was calculated for concatenated gene groups and individual genes at each nucleotide position. A sliding event (*Se*) score was calculated from this using a sliding window of five bases along the sequence, from which heatmaps were generated. To calculate the percentage of events greater than the average control promoter *Se* score (*d*), a 350-nucleotide region upstream of the estimated TATA box region was analysed where the total number of *Se* scores exceeding the control group average sliding event score (*Se* ^*C*^) was divided by the total number of events in that region (*N*).

$${d} = \frac{\text{Total number of}\ Se\ \text{events where}\ Se\ >\ Se^{C}}{N} \times 100$$

To calculate the promoter region *d* scores from PhyloP data, the total number of PhyloP (*p*) scores in the 350-nucleotide regions less than the control group average promoter region (*p* ^*C*^) took the place of the total number of *Se* events where *Se* is greater than *Se* ^*C*^ (see equation below).

$$d^{P} = \frac{\text{Total number of}\ p\ \text{scores}\ <\ p^{C}}{N} \times 100$$

Significance was calculated using non-parametric chi-squared tests compared to the control group *d* score. Significance values where *p* ≤ 0.05 with FDR or Bonferroni correction were considered statistically significant.

### Identification of somatic and germ cell-specifically expressed genes in *D. melanogaster*

We defined somatic and germline genes using several steps. First, using processed RNA-seq data from vasa-GFP ± cells FACS-sorted from ovaries of transgenic vas-GFP *Drosophila melanogaster* strain (w[*]; TI{TI}vas[AID:EGFP]) [45], we defined germline-enriched (vasa-GFP +) using log_2_ fold change > 2, adjusted *p* value < 0.01 parameters and soma-enriched (vasa-GFP-) genes using log_2_ fold change < -1, adjusted *p* value < 0.01 parameters (Figs. 5A and 5B). Next, using processed RNA-seq data from fly ovaries (modENCODE), we filtered genes that are expressed in adult fly ovaries using RPKM > 10 parameter for both soma and germline genes. To stringently define the germline genes, we additionally used processed RNA-seq data from early fly embryos (0-2h, modENCODE; mRNA-seq fly embryo 0-2h; RPKM; mE_mRNA_em0-2h; FBlc0000086) to further filter the germline-enriched genes using > 9 RPKM parameter. To stringently define the somatic-specific genes, we additionally used processed RNA-seq data from ovarian somatic cells (OSCs) (*n* = 4, GSE160860**)** [46] and filtered the soma-enriched genes using > 20 RPKM to select genes that are expressed in OSCs as well as parameter < 2 RPKM for early fly embryo (0-2h, modENCODE) to filter out genes that are expressed in early embryos. This analysis generated a list of 107 germ cell-specifically expressed genes and of 59 somatic cell-specifically expressed genes (Fig. 5A and B, Supplementary Dataset 1).

### Comparative gene expression analysis between *D. melanogaster* and *D. yakuba*

Using publicly available raw RNA-seq data from *D. melanogaster* (*n* = 3) and *D. yakuba* (*n* = 4) ovaries, we trimmed adapter sequences from the raw reads using Cutadapt tool (v1.18, default parameters) and aligned the trimmed reads to the genome assemblies (dm6 and droYak2) using the RNA-seq aligner STAR (v2.7.3a). Gene counts were quantified using the featureCounts tool (Subread package v1.5.3). Orthologues genes were identified using blastDm2FB in UCSC Table Browser. Gene count normalization and differential gene expression analysis between the species were performed using DESeq2 (v1.30.1) with an additional species-specific gene lengths normalization step.

### Motif enrichment analysis

The SEA tool in MEME-suite (version 5.5.5) was used to identify significantly enriched motifs in 350-nucleotide promoter regions [71, 72]. FlyAtlas2 RNA expression data from adult female ovaries was used to analyse expression of binding factors with enriched motifs [73, 74].

### Supplementary Information

Supplementary Material 1.

Supplementary Material 2.

## Data Availability

All data generated or analysed during this study are included in Supplementary Dataset 1.

## References

[1] Iwakawa HO, Tomari Y (2022). Life of RISC: Formation, action, and degradation of RNA-induced silencing complex. Mol Cell.

[2] Czech B, Munafò M, Ciabrelli F, Eastwood EL, Fabry MH, Kneuss E, Hannon GJ (2018). piRNA-guided genome defense: from biogenesis to silencing. Annu Rev Genet.

[3] Yamashiro H, Siomi MC (2018). PIWI-interacting RNA in Drosophila: biogenesis, transposon regulation, and beyond. Chem Rev.

[4] Slotkin RK, Martienssen R (2007). Transposable elements and the epigenetic regulation of the genome. Nat Rev Genet.

[5] Malone CD, Brennecke J, Dus M, Stark A, McCombie WR, Sachidanandam R, Hannon GJ (2009). Specialized piRNA pathways act in germline and somatic tissues of the drosophila ovary. Cell.

[6] Khurana JS, Theurkauf W (2010). piRNAs, transposon silencing, and Drosophila germline development. J Cell Biol.

[7] Senti K-A, Brennecke J (2010). The piRNA pathway: a fly's perspective on the guardian of the genome. Trends Genet.

[8] Sakakibara K, Siomi MC (2018). The PIWI-Interacting RNA molecular pathway: insights from cultured silkworm germline cells. BioEssays.

[9] Moelling K (2024). Epigenetics and transgenerational inheritance. J Physiol..

[10] Grentzinger T, Armenise C, Brun C, Mugat B, Serrano V, Pelisson A, Chambeyron S (2012). piRNA-mediated transgenerational inheritance of an acquired trait. Genome Res.

[11] Wang X, Ramat A, Simonelig M, Liu M-F (2023). Emerging roles and functional mechanisms of PIWI-interacting RNAs. Nat Rev Mol Cell Biol.

[12] Sato K, Siomi MC (2020). The piRNA pathway in Drosophila ovarian germ and somatic cells. Proc Jpn Acad Ser B.

[13] Signor S, Vedanayagam J, Kim BY, Wierzbicki F, Kofler R, Lai EC (2023). Rapid evolutionary diversification of the flamenco locus across simulans clade Drosophila species. PLoS Genet.

[14] Czech B, Hannon GJ (2016). One loop to rule them all: the ping-pong cycle and piRNA-guided silencing. Trends Biochem Sci.

[15] Haase AD (2022). An introduction to PIWI-interacting RNAs (piRNAs) in the context of metazoan small RNA silencing pathways. RNA Biol.

[16] Czech B, Preall JB, McGinn J, Hannon GJ (2013). A transcriptome-wide RNAi screen in the drosophila ovary reveals factors of the germline piRNA pathway. Mol Cell.

[17] Brown JS, Zhang D, Gaylord O, Chen W, Lee HC. Sensitized piRNA reporter identifies multiple RNA processing factors involved in piRNA-mediated gene silencing. Genetics. 2023;224(4):iyad095. 10.1093/genetics/iyad095 PMC10691750 37210214

[18] Handler D, Meixner K, Pizka M, Lauss K, Schmied C, Gruber FS, Brennecke J (2013). The genetic makeup of the drosophila piRNA pathway. Mol Cell.

[19] Munafò M, Lawless VR, Passera A, Macmillan S, Bornelöv S, Haussmann IU, Soller M, Hannon GJ, Czech B (2021). Channel nuclear pore complex subunits are required for transposon silencing in Drosophila. eLife.

[20] Nallasivan MP, Haussmann IU, Civetta A, Soller M (2021). Channel nuclear pore protein 54 directs sexual differentiation and neuronal wiring of female reproductive behaviors in Drosophila. BMC Biol.

[21] McQuarrie DWJ, Read AM, Stephens FHS, Civetta A, Soller M (2023). Indel driven rapid evolution of core nuclear pore protein gene promoters. Sci Rep.

[22] Haussmann IU, Hemani Y, Wijesekera T, Dauwalder B, Soller M (2013). Multiple pathways mediate the sex-peptide-regulated switch in female Drosophila reproductive behaviours. Proc Biol Sci.

[23] Tang S, Presgraves DC (2009). Evolution of the Drosophila nuclear pore complex results in multiple hybrid Incompatibilities. Science.

[24] Tang S, Presgraves DC (2015). Lineage-specific evolution of the complex Nup160 hybrid incompatibility between Drosophila melanogaster and its sister species. Genetics.

[25] Iwasaki YW, Siomi MC, Siomi H (2015). PIWI-interacting RNA: its biogenesis and functions. Annu Rev Biochem.

[26] Taylor MS, Kai C, Kawai J, Carninci P, Hayashizaki Y, Semple CAM (2006). Heterotachy in Mammalian promoter evolution. PLoS Genet.

[27] Balacco DL, Soller M (2019). The m(6)A writer: rise of a machine for growing tasks. Biochemistry.

[28] Bawankar P, Lence T, Paolantoni C, Haussmann IU, Kazlauskiene M, Jacob D, Heidelberger JB, Richter FM, Nallasivan MP, Morin V (2021). Hakai is required for stabilization of core components of the m6A mRNA methylation machinery. Nat Commun.

[29] van Lopik J, Alizada A, Trapotsi MA, Hannon GJ, Bornelöv S, Czech Nicholson B (2023). Unistrand piRNA clusters are an evolutionarily conserved mechanism to suppress endogenous retroviruses across the Drosophila genus. Nat Commun..

[30] Fridrich A, Moran Y (2023). Some flies do not play ping-pong. PLoS Biol.

[31] Chary S, Hayashi R. Mechanistic divergence of piRNA biogenesis in Drosophila. bioRxiv. 2022.11.14.516378.

[32] McDonald JH, Kreitman M (1991). Adaptive protein evolution at the Adh locus in Drosophila. Nature.

[33] Karolchik D (2004). The UCSC table browser data retrieval tool. Nucleic Acids Res.

[34] Kent WJ, Sugnet CW, Furey TS, Roskin KM, Pringle TH, Zahler AM, Haussler D (2002). The human genome browser at UCSC. Gen Res.

[35] Consortium GO (2021). The gene ontology resource: enriching a GOld mine. Nucleic Acids Res.

[36] Ashburner M, Ball CA, Blake JA, Botstein D, Butler H, Cherry JM, Davis AP, Dolinski K, Dwight SS, Eppig JT (2000). Gene ontology: tool for the unification of biology. The gene ontology consortium. Nat Genet.

[37] Mi H, Muruganujan A, Ebert D, Huang X, Thomas PD (2018). PANTHER version 14: more genomes, a new PANTHER GO-slim and improvements in enrichment analysis tools. Nucleic Acids Res.

[38] Sanfilippo P, Wen J, Lai EC (2017). Landscape and evolution of tissue-specific alternative polyadenylation across Drosophila species. Gen Biol.

[39] Smolko AE, Shapiro-Kulnane L, Salz HK (2018). The H3K9 methyltransferase SETDB1 maintains female identity in Drosophila germ cells. Nat Commun.

[40] Vankuren NW, Vibranovski MD (2014). A novel dataset for identifying sex-biased genes in Drosophila. J Genomics.

[41] Hart CM, Cuvier O, Laemmli UK (1999). Evidence for an antagonistic relationship between the boundary element-associated factor BEAF and the transcription factor DREF. Chromosoma.

[42] Yang J, Ramos E, Corces VG (2012). The BEAF-32 insulator coordinates genome organization and function during the evolution of Drosophila species. Genome Res.

[43] Sawado T, Hirose F, Takahashi Y, Sasaki T, Shinomiya T, Sakaguchi K, Matsukage A, Yamaguchi M (1998). The DNA replication-related element (DRE)/DRE-binding factor system is a transcriptional regulator of the Drosophila E2FGene*. J Biol Chem.

[44] Kim J, Johnson K, Chen HJ, Carroll S, Laughon A (1997). Drosophila Mad binds to DNA and directly mediates activation of vestigial by Decapentaplegic. Nature.

[45] Alizada A, Hannon GJ, Nicholson BC. Ovo is a master regulator of the piRNA pathway in animal ovarian germ cells. bioRxiv. 2024.04.23.590802.

[46] Eastwood EL, Jara KA, Bornelöv S, Munafò M, Frantzis V, Kneuss E, Barbar EJ, Czech B, Hannon GJ (2021). Dimerisation of the PICTS complex via LC8/Cut-up drives co-transcriptional transposon silencing in Drosophila. eLife.

[47] Treiber CD, Waddell S (2020). Transposon expression in the Drosophila brain is driven by neighboring genes and diversifies the neural transcriptome. Genome Res.

[48] Laverty C, Lucci J, Akhtar A (2010). The MSL complex: X chromosome and beyond. Curr Opin Genet Dev.

[49] Straub T, Becker PB (2007). Dosage compensation: the beginning and end of generalization. Nat Rev Genet.

[50] Brennecke J, Malone CD, Aravin AA, Sachidanandam R, Stark A, Hannon GJ (2008). An epigenetic role for maternally inherited piRNAs in transposon silencing. Science.

[51] Luo Y, He P, Kanrar N, Fejes Toth K, Aravin AA (2023). Maternally inherited siRNAs initiate piRNA cluster formation. Mol Cell.

[52] Parhad SS, Yu T, Zhang G, Rice NP, Weng Z, Theurkauf WE (2020). Adaptive evolution targets a piRNA precursor transcription network. Cell Rep.

[53] Parhad SS, Tu S, Weng Z, Theurkauf WE (2017). Adaptive evolution leads to cross-species incompatibility in the piRNA transposon silencing machinery. Dev Cell.

[54] Kelleher ES, Edelman NB, Barbash DA (2012). Drosophila interspecific hybrids phenocopy piRNA-pathway mutants. PLoS Biol.

[55] Vermaak D, Henikoff S, Malik HS (2005). Positive selection drives the evolution of rhino, a member of the heterochromatin protein 1 family in drosophila. PLoS Genet.

[56] Obbard DJ, Finnegan DJ (2008). RNA interference: endogenous siRNAs derived from transposable elements. Curr Biol.

[57] Blumenstiel JP, Erwin AA, Hemmer LW (2016). What drives positive selection in the drosophila piRNA machinery? The genomic autoimmunity hypothesis. Yale J Biol Med.

[58] Wang L, Barbash DA, Kelleher ES (2020). Adaptive evolution among cytoplasmic piRNA proteins leads to decreased genomic auto-immunity. PLoS Genet.

[59] Parhad SS, Theurkauf WE (2019). Rapid evolution and conserved function of the piRNA pathway. Open Biol.

[60] Lawlor MA, Ellison CE (2023). Evolutionary dynamics between transposable elements and their host genomes: mechanisms of suppression and escape. Curr Opin Genet Dev.

[61] Soller M, Haussmann IU, Hollmann M, Choffat Y, White K, Kubli E, Schäfer MA (2006). Sex-peptide-regulated female sexual behavior requires a subset of ascending ventral nerve cord neurons. Curr Biol.

[62] Fan Y, Soller M, Flister S, Hollmann M, Müller M, Bello B, Egger B, White K, Schäfer MA, Reichert H (2005). The egghead gene is required for compartmentalization in Drosophila optic lobe development. Dev Biol.

[63] Seeholzer LF, Seppo M, Stern DL, Ruta V (2018). Evolution of a central neural circuit underlies Drosophila mate preferences. Nature.

[64] Main BJ, Smith AD, Jang H, Nuzhdin SV (2013). Transcription start site evolution in Drosophila. Mol Biol Evol.

[65] Tamura K, Stecher G, Kumar S (2021). MEGA11: molecular evolutionary genetics analysis version 11. Mol Biol Evol.

[66] Lack JB, Cardeno CM, Crepeau MW, Taylor W, Corbett-Detig RB, Stevens KA, Langley CH, Pool JE (2015). The Drosophila genome nexus: a population genomic resource of 623 Drosophila melanogaster genomes, including 197 from a single ancestral range population. Genetics.

[67] Lack JB, Lange JD, Tang AD, Corbett-Detig RB, Pool JE (2016). A thousand fly genomes: an expanded Drosophila genome nexus. Mol Biol Evol.

[68] Mackay TFC, Richards S, Stone EA, Barbadilla A, Ayroles JF, Zhu D, Casillas S, Han Y, Magwire MM, Cridland JM (2012). The Drosophila melanogaster genetic reference panel. Nature.

[69] Gramates LS, Agapite J, Attrill H, Calvi BR, Crosby MA, dos Santos G, Goodman JL, Goutte-Gattat D, Jenkins VK, Kaufman T (2022). FlyBase: a guided tour of highlighted features. Genetics.

[70] Tang H, Lewontin RC (1999). Locating regions of differential variability in DNA and protein sequences. Genetics.

[71] Bailey TL, Grant CE. SEA: simple enrichment analysis of motifs. bioRxiv. 2021.08.23.457422.

[72] Bailey TL, Johnson J, Grant CE, Noble WS (2015). The MEME suite. Nucleic Acids Res.

[73] Yanai I, Benjamin H, Shmoish M, Chalifa-Caspi V, Shklar M, Ophir R, Bar-Even A, Horn-Saban S, Safran M, Domany E (2004). Genome-wide midrange transcription profiles reveal expression level relationships in human tissue specification. Bioinformatics.

[74] Leader DP, Krause SA, Pandit A, Davies SA, Dow JAT (2017). FlyAtlas 2: a new version of the Drosophila melanogaster expression atlas with RNA-Seq, miRNA-Seq and sex-specific data. Nucleic Acids Res.

