# Supplementary material for: Rapid evolution of promoters from germline-specifically expressed genes including transposon silencing factors
Source: BMC Genomics. 2024 Jul 8;25:678. doi: 10.1186/s12864-024-10584-9 (PMC11229233; doi:10.1186/s12864-024-10584-9)

Supplementary Materials

Rapid evolution of promoters from germline-specifically expressed genes including transposon silencing factors

DAVID W. J. MCQUARRIE[1,2], AZAD ALIZADA[3], BENJAMIN CZECH NICHOLSON[3] AND MATTHIAS SOLLER[1,2*]

Supplementary Fig. 1: Germ cell-specific piRNA factors are under positive selection and accumulate rapid sequence changes in their promoters

A) Individual gene sequence change, indel, and base change *d* scores and statistically significant accumulation of sequence changes for the somatic piRNA factors and germ cell-specific piRNA factors. Statistically significant differences from non-parametric chi-squared tests are indicated by asterisks (* $p \leq 0.05$, ** $p \leq 0.01$, *** $p \leq 0.001$, **** $p \leq 0.0001$ following FDR correction).

B) Evolution of coding regions analysed by MKT tests for polymorphisms and divergence between *D. melanogaster* and *D. simulans* for core genes in the somatic and germ cell transposon silencing pathways. R, replacement; S, synonymous. Statistically significant differences from Fisher's Exact Tests are indicated by asterisks (**** $p \leq 0.0001$ following FDR correction).

Supplementary Fig. 2: Genes coding for members of a subgroup of protein complexes implicated in piRNA silencing accumulate sequence changes in their promoters

Individual gene *d* scores and statistically significant accumulation of sequence changes for each gene separated by protein complex compared to the somatic piRNA factors. Statistically significant differences from non-parametric chi-squared tests are indicated by asterisks (* $p \leq 0.05$, ** $p \leq 0.01$, **** $p \leq 0.0001$ following Bonferroni correction).

Supplementary Fig. 3: Promoter evolution of protein complex genes involved in germ cell transposon silencing

A and C) Heatmaps indicating indel (A, blue) or base change (C, green) accumulation in protein complex genes involved in germ cell transposon silencing compared to somatic and germ cell piRNA factor group genes among closely related *D. melanogaster*, *D. simulans*, *D. sechellia*, *D. yakuba* and *D. erecta*. Regions of 1000 nucleotides upstream and 300 nucleotides downstream of the TSS were analysed. The blue line indicates the promoter region used for quantification of the substitution rate.

B and D) Comparison of indel (B, blue) or base change (D, green) *d* scores for each of the protein complex gene groups compared to the somatic and germ cell piRNA factors. Statistically significant differences from non-parametric chi-squared tests are indicated by asterisks (* $p \leq 0.05$, ** $p \leq 0.01$, *** $p \leq 0.001$, **** $p \leq 0.0001$ following Bonferroni correction).

E) Individual gene *d* scores and statistically significant accumulation of indels or base changes for each gene separated by protein complex compared to the somatic and germ cell piRNA factors. Comparison of indel (B, blue) or base change (D, green) *d* scores for each of the protein complex gene groups compared to the control group. Statistically significant differences from non-parametric chi-squared tests are indicated by asterisks (* $p \leq 0.05$, ** $p \leq 0.01$, *** $p \leq 0.001$, **** $p \leq 0.0001$ following Bonferroni correction).

Supplementary Fig. 4: Coding sequences evolution of protein complex genes involved in germ cell transposon silencing.

A) Impact of protein complex genes involved in germ cell transposon silencing on transposon de-repression. Unprocessed data were taken from Czech et al. (2013) [1]. Derepression scores (*z*) were concatenated for protein complexes in each transposon type (HeTA, TAHRE, blood, burdock) and an average of all.

B) Evolution of coding regions analysed by MKT tests for polymorphisms and divergence between *D. melanogaster* and *D. simulans* for individual genes from the selected germline transposon silencing implicated protein complexes. R, replacement; S, synonymous. Statistically significant differences from Fisher's Exact Tests are indicated by asterisks (* $p \leq 0.05$, ** $p \leq 0.01$, *** $p \leq 0.001$, **** $p \leq 0.0001$ following FDR correction).

Supplementary Fig. 5: Motif enrichment analysis of piRNA factors.

Enriched motifs are listed for three gene groups (somatic and germ cell piRNA factors, germ cell-specific piRNA factors, and the FEPG gene group) according to their motif rank for individual DNA binding factors (motif ID and consensus), listing the percentage of true positives (TP, number of times a motif is present in the given primary sequences), significance (corrected p values), and normalised expression (FPKM) from adult ovaries.

Supplementary Fig. 6: Distribution of expression changes of germ cell and somatic cell-specifically expressed genes.

Comparison of the significant expression distribution for $\log_2$ fold change in *D. melanogaster* (>0.5) and *D. yakuba* (<-0.5) expression separated into PhyloP conservation promoter score (d^P) ranges >25-50, >50-75, and >75-100. Expression changes for germ cell-specific and somatic cell-specific gene groups were analysed between *D. melanogaster* and *D. yakuba*. The

median line is shown within each box. Outliers have been removed from the figure. Mann Whitney U tests were used to calculate significance (* $p \leq 0.05$ following FDR correction).

Supplementary References

1. Czech B, Preall J, B. , McGinn J, Hannon G, J. : **A Transcriptome-wide RNAi Screen in the Drosophila Ovary Reveals Factors of the Germline piRNA Pathway**. *Molecular Cell* 2013, **50**(5):749-761.

Supplementary Figure 1

A

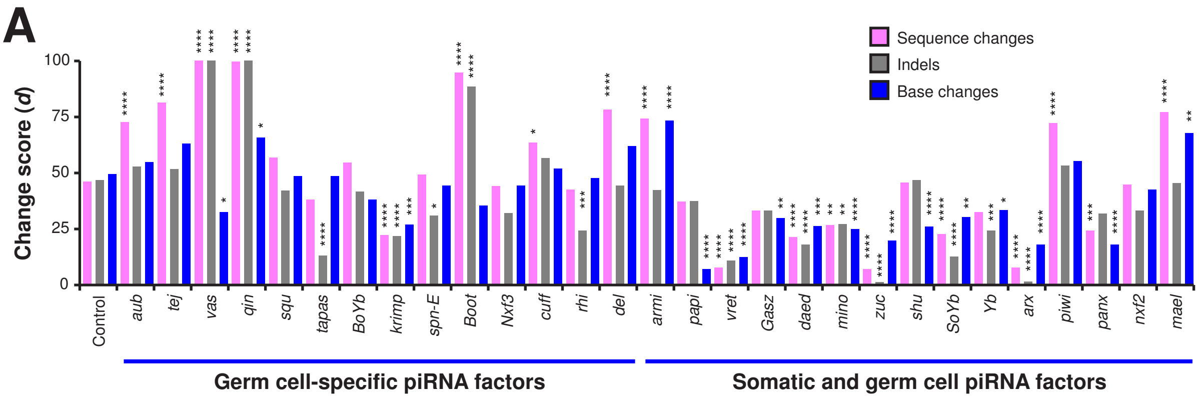

B

	Population	Polymorphism R	Polymorphism S	Polymorphism R/S	Divergence R	Divergence S	Divergence R/S	Fisher's exact *P*-value (FDR)	
Germ cell-specific piRNA factors	Congo	133	102	1.304	1271	519	2.449	0.000023	****
	Zambia	645	384	1.680	1204	473	2.545	0.000004	****
Somatic and germ cell piRNA factors	Congo	149	97	1.536	571	400	1.428	0.663171	
	Zambia	588	446	1.318	530	338	1.568	0.090960	

Supplementary Figure 2

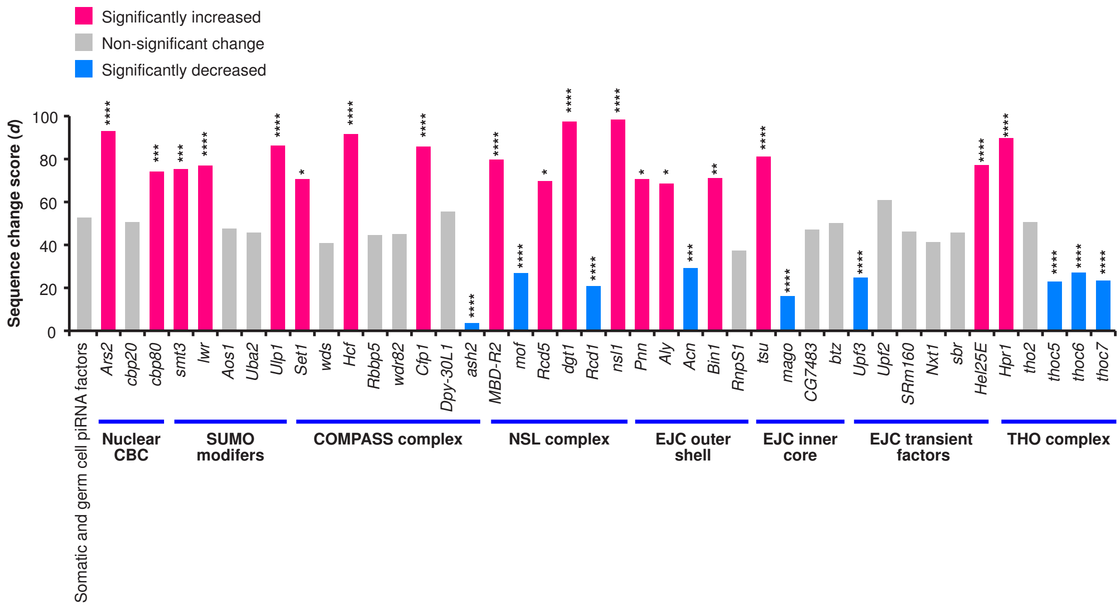

Supplementary Figure 3

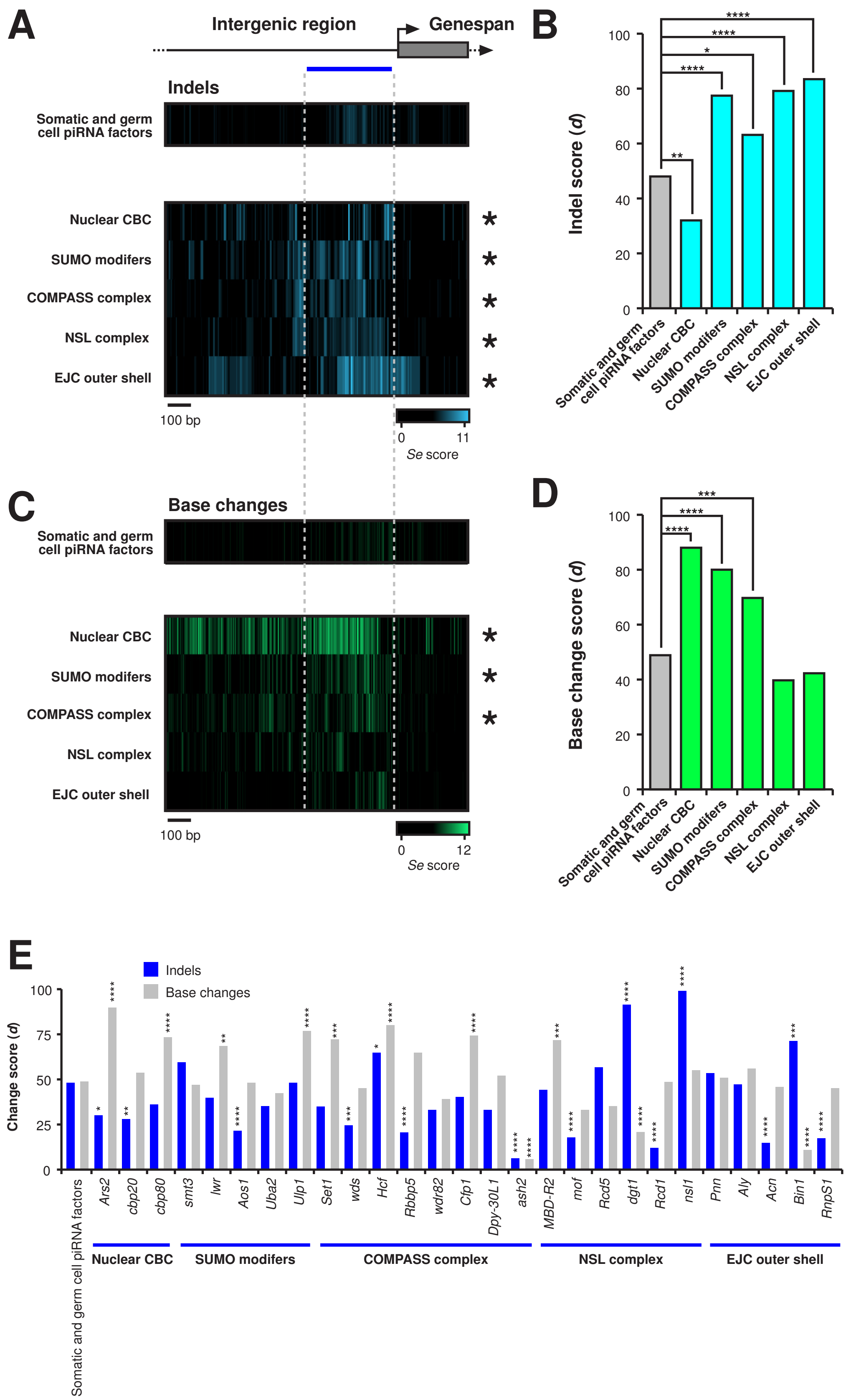

Supplementary Figure 4

A

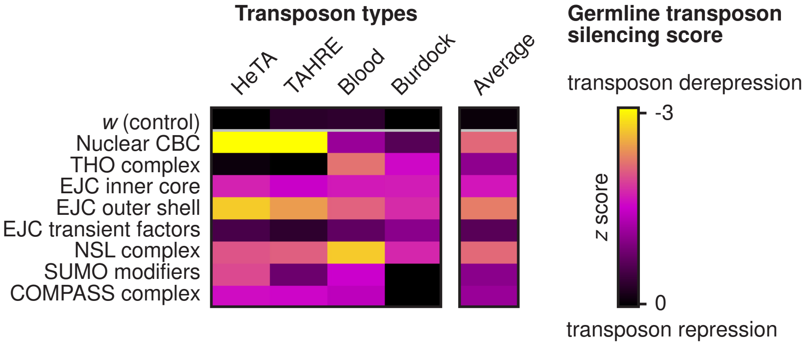

B

	Population	Polymorphism R	Polymorphism S	Polymorphism R/S	Divergence R	Divergence S	Divergence R/S	Fisher's exact *P*-value (FDR)	
Nuclear CBC	Congo	9	20	0.450	86	106	0.811	0.330182	
	Zambia	25	67	0.373	85	98	0.867	0.005210	**
THO complex	Congo	24	29	0.828	77	124	0.621	0.573867	
	Zambia	102	136	0.750	70	108	0.648	0.594249	
EJC inner core	Congo	7	11	0.636	26	52	0.500	0.895886	
	Zambia	32	68	0.471	24	46	0.522	0.926331	
EJC outer shell	Congo	15	26	0.577	274	135	2.030	0.000533	***
	Zambia	64	74	0.865	268	127	2.110	0.000042	****
EJC transient factors	Congo	18	66	0.273	110	124	0.887	0.000157	***
	Zambia	100	259	0.386	100	103	0.971	0.000003	****
NSL complex	Congo	42	62	0.677	366	253	1.447	0.000914	***
	Zambia	206	321	0.642	345	220	1.568	7.44E-12	****
SUMO modifiers	Congo	17	19	0.895	349	147	2.374	0.014222	*
	Zambia	94	97	0.969	337	133	2.534	0.000001	****
COMPASS complex	Congo	6	41	0.146	32	90	0.356	0.106720	
	Zambia	47	124	0.379	29	73	0.397	0.889564	

Supplementary Figure 5

Group	Rank	Motif ID	Consensus	TP (%)	p value (FDR)	FPKM (adult ovary)
Somatic and germ cell piRNA factors	1	jim	AAAAAAAAMSA	47	3.16E-03	1.98
	2	Cf2	TADSYGAAA	60	5.19E-03	6.38
Germ cell-specific piRNA factors	1	sna	KBBKKRACAGGTGG	50	2.90E-03	0.00
	2	sna	BKKRACAGGTG	50	2.90E-03	0.00
	3	fkh	CTTTTGTAAAYA	93	4.42E-03	0.01
	4	Pph13	YTAATTR	100	5.79E-03	0.01
FEPG	1	ato	MCACMTGWCRC	27	1.05E-04	0.00
	2	Mad	KCYGSYGKCGCSBC	24	2.44E-04	16.40
	3	BEAF-32	TATCGATA	31	3.43E-04	23.47
	4	Dref	ATAWCGATAR	33	7.95E-04	12.41
	5	CG33557	MCASATGKHRHVVMNAYWAMA	98	1.15E-03	0.64
	6	brk	KYRGCGCCASBHAV	64	1.24E-03	0.66
	7	klu	KGYGKGGGTGKKDBN	60	1.57E-03	0.45
	8	brk	SYGGCGCY	51	1.65E-03	0.66
	9	ERR	MAAGGTCA	27	1.66E-03	2.84
	10	Dref	WNWTATCGATA	27	1.66E-03	12.41
	11	net	RCCACCTGK	71	2.08E-03	0.17
	12	amos	RMCATCTGBCV	38	3.37E-03	0.00
	13	klu	YGKGGGTGKKD	47	4.07E-03	0.45
	14	Hr83	AAAGTCAMMVTRG	40	4.77E-03	0.00
	15	Dref	WNWTATCGATAR	76	4.96E-03	12.41
	16	Hr83	AAAGTCAMMVTRGM	31	5.85E-03	0.00
	17	Antp	TTTTAATKA	16	6.07E-03	0.12
	18	klu	TGYGKGGGTGK	16	6.07E-03	0.45
	19	net	RCCACCTGK	78	6.71E-03	0.17

Supplementary Figure 6

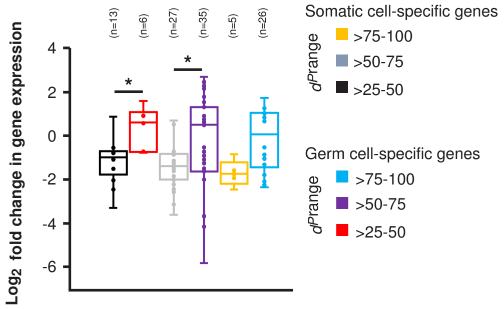

Supplement: Supplementary file 1 — Supplementary Material 1. [file 12864_2024_10584_MOESM1_ESM.pdf]
